# The Effect of Carbon Black on the Properties of Plasticised Wheat Gluten Biopolymer

**DOI:** 10.3390/molecules25102279

**Published:** 2020-05-12

**Authors:** Oisik Das, Antonio J Capezza, Julia Mårtensson, Yu Dong, Rasoul Esmaeely Neisiany, Leonardo Pelcastre, Lin Jiang, Qiang Xu, Richard T. Olsson, Mikael S Hedenqvist

**Affiliations:** 1 Department of Engineering Sciences and Mathematics, Luleå University of Technology, 97 187 Luleå, Sweden; leonardo.pelcastre@ltu.se; 2 Department of Fibre and Polymer Technology, School of Engineering Sciences in Chemistry, Biotechnology and Health, KTH Royal Institute of Technology, SE-100 44 Stockholm, Sweden; ajcv@kth.se (A.J.C.); julia.grimmie@gmail.com (J.M.); rols@kth.se (R.T.O.); 3 Department of Plant Breeding, Faculty of Landscape Planning, Horticulture and Crop Production Sciences, Swedish University of Agricultural Sciences, 23053 Alnarp, Sweden; 4 School of Civil and Mechanical Engineering, Curtin University, Perth WA 6845, Australia; Y.Dong@curtin.edu.au; 5 Department of Materials and Polymer Engineering, Faculty of Engineering, Hakim Sabzevari University, Sabzevar 9617976487, Iran; r.esmaeely@hsu.ac.ir; 6 School of Mechanical Engineering, Nanjing University of Science and Technology, Nanjing 210094, China; ljiang@njust.edu.cn (L.J.); xuqiang@njust.edu.cn (Q.X.)

**Keywords:** gluten, carbon black, biocomposites, plasticisers, tensile properties, fire resistance

## Abstract

Wheat gluten biopolymers generally become excessively rigid when processed without plasticisers, while the use of plasticisers, on the other hand, can deteriorate their mechanical properties. As such, this study investigated the effect of carbon black (CB) as a filler into glycerol-plasticised gluten to prepare gluten/CB biocomposites in order to eliminate the aforementioned drawback. Thus, biocomposites were manufactured using compression moulding followed by the determination of their mechanical, morphological, and chemical properties. The filler content of 4 wt% was found to be optimal for achieving increased tensile strength by 24%, and tensile modulus by 268% along with the toughness retention based on energy at break when compared with those of glycerol-plasticised gluten. When reaching the filler content up to 6 wt%, the tensile properties were found to be worsened, which can be ascribed to excessive agglomeration of carbon black at the high content levels within gluten matrices. Based on infrared spectroscopy, the results demonstrate an increased amount of β-sheets, suggesting the formation of more aggregated protein networks induced by increasing the filler contents. However, the addition of fillers did not improve fire and water resistance in such bionanocomposites owing to the high blend ratio of plasticiser to gluten.

## 1. Introduction

Plastics derived from natural resources offer great interest to material scientists and engineers due to a more limited environmental impact compared with conventional petroleum-based counterparts [1]. Here, a variety of protein-based polymers such as soy, gluten, and milk proteins have emerged in the past few decades because of their large-scaled availability, renewability, biodegradability, and cost-effectiveness [2,3]. Among some other plant proteins, such as corn and soy proteins, wheat gluten (WG) can be obtained as an agricultural by/co-product from wheat and cereal production industries [4]. WG is a particularly promising substitute for petroleum-based plastics since it has excellent barrier properties when exposed to oxygen/carbon dioxide in dry conditions [5], a good potential of film formability [6] and rapid biodegradation rates in nature. Nonetheless, without using any plasticiser (or with only lower loading amounts), WG processing at high-temperature levels yields a stiff and hard material [7] owing to its higher cross-link density, and thus may limit its widespread applicability.

To reduce the brittleness of WG after processing, plasticisers (e.g., glycerol) are generally utilised despite the possible detrimental role of plasticisers to mechanical properties of WG. There have been some studies attempting to enhance the performance properties of WG by adding reinforcing fibres and particulates. Song et al. [8] developed WG biocomposites with the addition of hydroxyethyl cellulose and glycerol plasticiser. It was reported that the inclusion of the filler was beneficial for increasing both the tensile strength and tensile modulus of corresponding composites. More recently, Yang et al. [9] manufactured WG bionanocomposites reinforced with lignin nanoparticles. It was observed that the addition of lignin nanoparticles resulted in an enhancement of tensile strength and tensile modulus along with water uptake resistance. Surprisingly, with only 3 wt% of lignin nanoparticles, the tensile strength increased to a value 2.4 times higher than without the particles. At the same time, the modulus increased to a value three times higher. Elsewhere, Hemsri et al. [10] investigated the effect of silane-treated coconut fibres on WG biocomposites. A significant increase in mechanical properties was manifested due to the better interfacial bonding between treated fibres and WG matrices. In recent years, carbon-based materials have been employed as reinforcing fillers in biocomposites [11,12,13,14,15] due to their porous nature, high inherent hardness/modulus, and fire resistance. In particular, carbonaceous biochars were added to WG to create fully bio-based composites [16]. The biochars enabled an increase in both mechanical properties and water resistance of WG polymer. However, the aforementioned study was conducted on a rigid unplasticised WG polymer.

For WG to be useable in packaging applications, the material should possess good flexibility (at reduced brittleness), which can be achieved by mixing plasticisers into the WG matrix. Furthermore, carbon-based fillers promote the stiffness or rigidity of resulting composites due to their high inherent modulus. The modulus of pine-based biochars via nanoindentation was reported to be 4.6 GPa as opposed to only 0.16 GPa for neat WG [16]. Carbon-based fillers can thus provide the resistance to reduce the deformability of surrounding matrices, thus increasing the elastic modulus of the entire composite system [15]. WG, without any plasticiser, yields a very stiff but strong material after post-processing. The high-temperature (ca. 150 °C) processing creates a heat-induced cross-linking effect for the polymerisation of proteins with the rendered stiffness [17]. Overall, without using any plasticiser, processing WG inevitably yields a very stiff material while the use of plasticiser decreases the stiffness and tensile strength simultaneously. Consequently, it is necessary to understand the fundamental effect of carbon-based fillers in flexible plasticised WG composites, in order to balance the final properties of the materials towards their applications.

The aim of this study was to evaluate the effect of carbon black (CB) as fillers on the performance-based properties of plasticised WG composites. In particular, CB fillers were added to alleviate the issue of mechanical property loss due to plasticiser addition in gluten biopolymer. This study is different from the Wu et al. [18] investigation because the focus is not on polymer conductivity for electrical applications. Tensile properties, morphological structures, fire-resistance, water uptake, and chemical properties of such resulting composites at the CB contents of 2, 4, and 6 wt% were determined. The CB content was limited up to 6 wt% because excessive amounts beyond this level would give rise to typical filler agglomeration resulting in poor material properties, as evidenced elsewhere [15]. It is anticipated that well-tailored WG/CB biocomposites can be produced for targeting material packaging applications with effective environmental sustainability.

## 2. Materials and Methods 

### 2.1. Material Manufacturing 

WG powders were supplied by Lantmännen Reppe AB, Sweden at a gluten protein content of 78 wt% (nitrogen conversion factor: 5.7). The remaining composition consists of 5.8 wt% starch, 1.2 wt% fats, and 0.9 wt% inorganic ashes of dry weight (moisture content of total weight: 6.9 wt%). Glycerol as the plasticiser in this study was purchased from Karlshamn Tefac AB, Sweden. Carbon black Printex XE 2B was supplied by Orion Engineered Carbons, USA with an average particle size of 30 nm and surface area of 1000 m^2^/g. Material preparation was undertaken by initially mixing 25 wt% glycerol with WG using a stationary immersion blender for 20 min. Subsequently, the mixtures were kept at room temperature for 12 h and then flash frozen by using liquid N_2_, which was followed by grinding (Ultra Centrifugal Mill ZM 200, Retsch GmBH, 5657 HAAN, Germany). WG powders were further mixed with CB at different contents in a blender for 30 min prior to the final compression moulding process (Fortijne Presses TP 400, The Netherlands) to prepare gluten/CB biocomposites. A hot press was employed with a compressive force of 250 kN at 140 °C for 20 min to manufacture square material samples with dimensions of 100 × 100 × 0.5 mm. These samples were conditioned at 23 ± 1℃ with a relative humidity of 50% ± 2.5% for 48 h before further testing and characterisation. The detailed blend ratios of different material samples are indicated in Table 1. 

### 2.2. Material Characterisation

The tensile properties of gluten/CB biocomposites in terms of tensile strength, Young’s modulus and elongation at break were determined on an Instron 5566 Universal Testing Machine (Instron, Double column, Norwood, MA, USA) at a crosshead speed of 50 mm/min with a load cell of 500 N according to ASTM D638. Before the tests, all the specimens, which were cut into dog bone shapes having a width of 12.7 mm, were conditioned at 23 ± 1 °C and 50% ± 2.5% relative humidity for 48 h. The tensile tests were conducted at the same temperature and humidity. For each material batch, at least five replicate tests were performed with reported average data and associated standard deviations for test reproducibility.

The morphological structures of tensile fractured samples were investigated on a scanning electron microscope (SEM) (Hitachi TM 100, Tokyo, Japan) at an accelerating voltage of 10 kV with a working distance of 6 mm. The vertical burn tests were carried out in accordance with ASTM D3801 (equivalent to UL-94). The water absorption was measured by immersing the samples in distilled water for 48 h to determine the weight difference before and after the tests. The Fourier Transform-Infrared (FT-IR) spectra were recorded in ATR mode on a Perkin Elmer Spectrum 400 instrument equipped with a single reflection ATR accessory (Golden Gate) from Graseby Specac, Orpington, UK. The samples were placed over the crystal to test the characteristic bands. For all samples, 16 scans were conducted in a wavenumber range of 600–4000 cm^−1^ with a resolution of 4 cm^−1^. A curve deconvolution was performed on all FT-IR spectra using Perkin Elmer Software with an enhancement factor and a smoothing filter of 2% and 70%, respectively. 

### 2.3. Analysis of Variance (ANOVA)

A single factor ANOVA (α = 0.05) was performed with the response to tensile strength, modulus, and elongation at break [19]. An appropriate post hoc test (Scheffe) was performed in case the null hypothesis was rejected.

## 3. Results and Discussion

### 3.1. Tensile Properties

**Figure 1:**
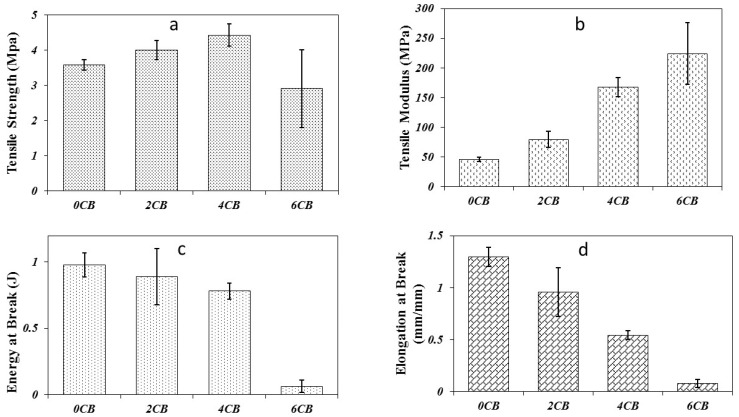
Tensile properties of gluten/CB biocomposites, (**a**) Tensile strength, (**b**) Tensile modulus, (**c**) Energy at Break, and (**d**) Elongation at break.

**Figure 2:**
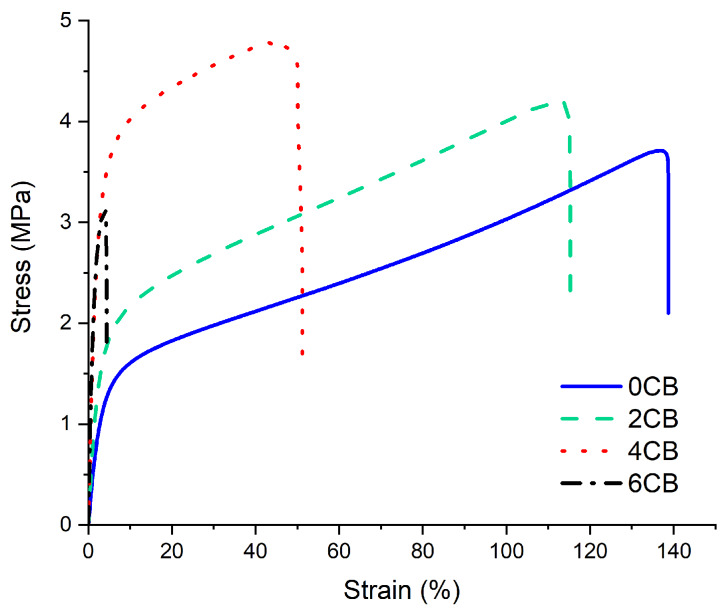
Stress vs. strain curves of gluten/CB biocomposites.

The tensile properties of different samples are presented in Figure 1. The stress vs. strain curves are shown in Figure 2. In general, tensile strengths of all the samples are quite low (ca. 3–4.5 MPa), as shown in Figure 1a, which can be attributed to the presence of glycerol in the composite. However, the strength increases moderately with the increasing CB content up to 4 wt%, where 4CB sample had the highest tensile strength at ca. 4.4 MPa. From a statistical point of view, 4CB was detected to be significantly higher than 6CB only in tensile strength, which was similar to those of 0CB and 2CB. However, 6CB demonstrates statistically comparable tensile strength to those of 0CB and 2CB, as displayed in Table 2. Nevertheless, the decrease in the tensile strength for the 6CB sample can be attributed to the large amount of CB that aggregated to cause tensile failure through an increase in stress concentration sites. In spite of being statistically insignificant, the addition of 4 wt% CB was still found to increase the tensile strength of neat WG by ca. 24%. 

Figure 1b shows that Young’s modulus of all biocomposite samples is higher than that of neat WG (0CB). From Table 3, all samples except 2CB reveal significantly higher moduli than that of 0CB albeit no statistical difference was shown for 4CB and 6CB. The increasing moduli with rising CB content in gluten/CB biocomposites is expected, owing to the presence of these rigid fillers [20]. The incorporation of CB particles into WG reduces the mobility of biopolymeric molecule chains consequently enhancing the stiffness of resulting biocomposites. As illustrated in Figure 1c, the toughness (i.e., energy at break) of biocomposites declined with increasing CB contents, especially reaching the lowest level for 6CB due to the possible agglomeration that initiated crack failure. The elongation at break also demonstrates a similar trend, as depicted in Figure 1d and Figure 2. However, statistical analysis suggested that the toughness values of 0CB, 2CB, and 4CB could be insignificantly different according to Table 4. Conversely, the decrease in elongation at break appeared to be statistically significant with increasing CB contents according to Table 5. It is evidently shown that increasing the CB contents can make the resulting biocomposites less ductile while rendering them resistant to deformation.

### 3.2. Morphological Features

**Figure 3:**
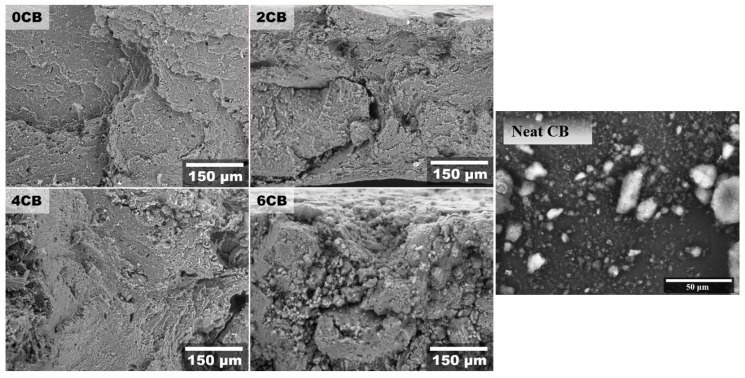
Scanning electron microscope (SEM) micrographs of tensile fractured gluten/CB biocomposites and neat CB.

Figure 3 exhibits the SEM images of neat CB and tensile fractured biocomposites. CB particles were observed to be in the form of particle agglomerates in various sizes, some of which are in the range of several tens of micrometres, indicating that embedded CBs can be categorised as micron-sized particles rather than nanoparticles with the average particle size of 30 nm. The SEM image of 0CB without any CB particles demonstrates typical ductile fracture behaviour of gluten without any relatively stiff inclusions. The fractured surfaces of 2CB and 4CB are comparable to that of 0CB as a result of statistically similar tensile strength levels shown in Figure 1A and Table 2. However, with increasing CB content, the fracture surface became more crenelated in nature with ridges and irregular cracks. The micrographs of 2CB and 4CB did not possess so many voids associated with particle debonding, signifying more effective stress transfer from WG matrices to CB to enhance their tensile strengths as depicted in Figure 1a. The SEM image of 6CB reveals irregular matrices with many cracks along with the CB agglomerates resulting in the reduction in tensile strength of biocomposites. The surface fracture of 6CB shows large aggregates of CB (> 150 µm), which can be associated with its poor tensile strength and high rigidity. The sample 4CB only showed scattered CB aggregates, thus correlating to its higher strength, when compared to 6CB. Thus, it is evident that the optimal percolation threshold in the WG composite is at a CB concentration of ca. 4 wt% (4CB). It is clearly shown that the threshold of 6 wt% CB should not be exceeded, otherwise the wettability of fillers in biopolymer matrices would be inadequate. Similar findings regarding carbon-based fillers were reported by previous studies [11,15]. 

### 3.3. Fire Resistance

**Figure 4:**
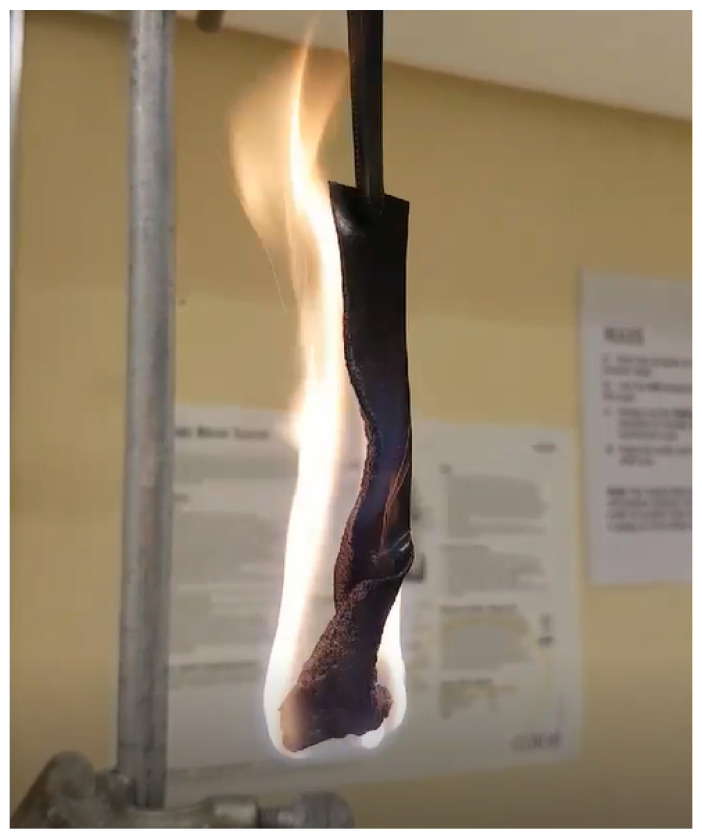
The burning behavior of a typical 2CB material sample.

The results from the vertical burn tests are summarised in Table 6. None of the tested samples were found to have self-extinguishing capabilities, thus resulting in no rating (NR) for all materials. All the samples ignited on the first flame application and burned all the way up to the holding clamp. When the samples were burned, it dripped flaming materials to ignite the cotton below. The samples failing to get a rating in the vertical burn test can be attributed to the presence of 25 wt% of glycerol which itself is combustible (flammability rating 1) (NCBI, PubChem Database. Glycerol). The higher amount of glycerol plasticiser at 25 wt% subdued any fire-resisting effect of CB, which was added at much lower amounts. The burning behaviour of the sample is illustrated in Figure 4.

### 3.4. Water Uptake Characteristics

The water uptake by the composite samples is presented in Table 7. Numerous factors govern the uptake of water into composite materials [21]. However, in the current composite system, the most likely factors influencing water uptake are the different abilities of the matrix and CB to absorb water, and the capillary action owing to bigger agglomerates at high filler contents [22]. Both WG and glycerol are hygroscopic in nature, whereas with increasing CB contents agglomerates form, constituting loosely connected particles which are porous, thus facilitating water absorption by a capillary effect. The CB inside the polymer matrix forms clusters, which according to Brosseau et al. [23] are secondary aggregates due to CB’s relatively high specific surface area of 1000 m^2^/g. When the samples were placed in contact with the compatible penetrant (in this study water), the liquid entered the holes and microvoids of these structures. Therefore, the entire composite system was prone to water uptake in this study, which is thus contrary to the previous results obtained by Ch’ng et al. [24] which showed a reduction in biodiesel uptake for CB within elastomer matrices in elastomer/CB composites. The lower liquid uptake with the higher loading of fillers is attributed to the barrier effect caused by CB. However, a plausible reason for the biodiesel to penetrate less was associated with its higher viscosity [25] as opposed to that of water used in this study.

### 3.5. FT-IR Analysis

**Figure 5:**
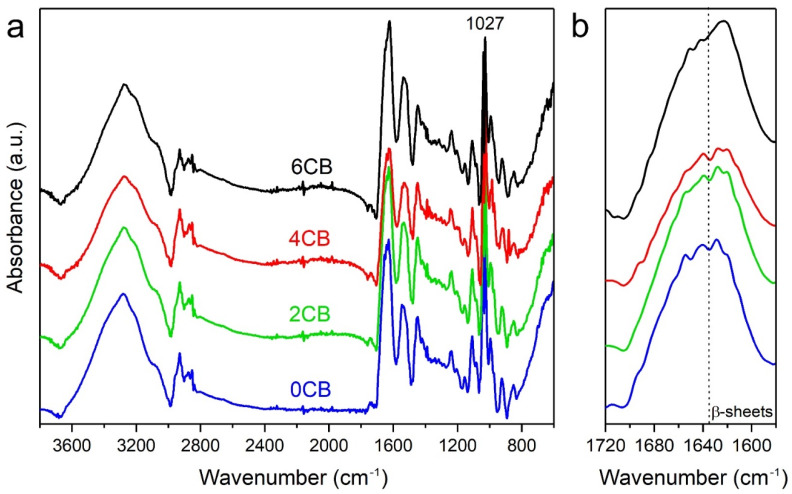
Fourier transform-infrared (FT-IR) spectra of gluten/CB biocomposites: (**a**) entire spectra and (**b**) enlarged amide region.

The FT-IR spectra of material samples are shown in Figure 5. No significant changes were found for biocomposites at different CB contents. The strong peak detected at 1027 cm^−1^ (Figure 5a) corresponds to 25 wt% glycerol contained within the composite samples [26]. Due to the absence of functional groups in CB, no new peaks appear in FT-IR spectra when combined with the gluten matrices [16]. The overall shape of the amide I region for the different composite samples (Figure 5b), was reported to be associated with conformational structures of proteins [27]. Here, an increase in the peak intensity for the β-sheet region in the wavenumber range of 1635–1580 cm^−1^ was observed among samples with increasing CB contents. The highest peak intensity detected for 6CB in Figure 5b can be mainly ascribed to more aggregated protein structures [16]. The shoulder from 1446 to 1416 cm^−1^ can be ascribed to -OH bending. This shoulder was lowest for 4CB and highest for 0CB. The former sample also had the lowest -OH interaction region (3600–3200 cm^−1^), in contrast to 0CB having stronger -OH interactions. The peak observed for 4CB at 880 cm^−1^ can be assigned to C=C bending in alkene, which could be either formed within the CB network or as the result of covalent bonding between CB and WG; this is more evident in the 4CB sample.

## 4. Conclusions

The present study attempted to investigate the effects of carbon black additions to plasticised WG biopolymer using glycerol. The biocomposites were holistically characterised for their mechanical properties, morphological structures, fire resistance, water uptake, and chemical properties. It was found that the tensile strength of biocomposites increased with increasing CB content up to 4 wt%, beyond which the strength level declined. It was suggested that the filler content threshold of ca. 4 wt% should be considered in such biocomposites since any excessive CB amounts would result in typical particle agglomeration, thus leading to more stress concentration sites to undermine tensile properties. Increasing the CB contents appeared to enhance the tensile moduli of biocomposites, but lower their toughness and elongation at break. Moreover, a higher amount of glycerol as a plasticiser in biocomposites tended to hinder fire resistance with all samples having no ratings in vertical burn tests. The porous secondary aggregate structures of CB enabled increased water uptake in the biocomposite samples, despite the formation of more aggregated protein structures. The effect of CB as a reinforcing filler was somehow undermined by using a large amount of plasticiser in biopolymer matrices. The future work should concentrate on optimal material formulation in relation to desirable content of plasticiser used for maximizing the use of carbon-based fillers.

## Figures and Tables

**Table 1:** The blend ratio of gluten/carbon black (CB) biocomposites.

Samples	WG (wt%)	Plasticiser (wt%)	CB (wt%)
0CB	75	25	0
2CB	73	25	2
4CB	71	25	4
6CB	69	25	6

**Table 2a:** 

Source of Variation	SS	Df	MS	F	*p*-Value	Fcrit
Between Groups	5.568	3	1.856	6.215	0.006	3.287
Within Groups	4.479	15	0.298			
Total	10.048	18				

From the above table, it can be observed that F > Fcrit. Hence, the null hypothesis is rejected. The tensile strength means of the samples are not all equal. At least one of the means is different. Therefore, the following post hoc test is conducted to identify where the difference lies.

**Table 2b:** 

Post Hoc	0CB	2CB	4CB
2CB	0.414		
4CB	0.845	0.430	
6CB	0.674	1.089	1.519

Coloured cells have significant mean differences.

**Table 3a:** 

Source of Variation	SS	df	MS	F	*p*-Value	Fcrit
Between Groups	90227.86	3	30075.95	45.690	0.000	3.287
Within Groups	9873.87	15	658.258			
Total	100101.7	18				

From the above table. it can be observed that F > Fcrit. Hence, the null hypothesis is rejected. The tensile modulus means of the samples are not all equal. At least one of the means is different. Therefore, the following post hoc test is conducted to identify where the difference lies.

**Table 3b:** 

Post Hoc	0CB	2CB	4CB
2CB	33.572		
4CB	121.475	87.902	
6CB	178.612	145.039	57.137

Coloured cells have significant mean differences.

**Table 4a:** 

Source of Variation	SS	df	MS	F	*p*-Value	Fcrit
Between Groups	2.224	3	0.741	47.344	0.000	3.287
Within Groups	0.234	15	0.015			
Total	2.459	18				

From the above table. it can be observed that F > Fcrit. Hence, the null hypothesis is rejected. The energy at break means of the samples are not all equal. At least one of the means is different. Therefore, the following post hoc test is conducted to identify where the difference lies.

**Table 4b:** 

Post Hoc	0CB	2CB	4CB
2CB	0.088		
4CB	0.195	0.106	
6CB	0.915	0.827	0.720

Coloured cells have significant mean differences.

**Table 5a:** 

Source of Variation	SS	df	MS	F	*p*-Value	Fcrit
Between Groups	3.718	3	1.239	69.547	0.000	3.287
Within Groups	0.267	15	0.017			
Total	3.985	18				

From the above table. it can be observed that F > Fcrit. Hence, the null hypothesis is rejected. The extension at break means of the samples are not all equal. At least one of the means is different. Therefore, the following post hoc test is done to identify where the difference lies.

**Table 5b:** 

Post Hoc	0CB	2CB	4CB
2CB	0.338		
4CB	0.751	0.412	
6CB	1.217	0.878	0.465

Coloured cells have significant mean differences.

**Table 6:** UL- 94 test data of gluten/CB biocomposites.

Samples	UL 94 Rating	Dripping
0CB	NR	Yes
2CB	NR	Yes
4CB	NR	Yes
6CB	NR	Yes

**Table 7:** Water uptake of gluten/CB biocomposites.

Samples	Water Sorption (wt% Increase)
0CB	26.91 ± 3.47
2CB	32.84 ± 0.68
4CB	35.95 ± 0.22
6CB	41.51 ± 0.83
